# Human C1q Induces Apoptosis in an Ovarian Cancer Cell Line *via* Tumor Necrosis Factor Pathway

**DOI:** 10.3389/fimmu.2016.00599

**Published:** 2016-12-21

**Authors:** Anuvinder Kaur, Sami H. A. Sultan, Valarmathy Murugaiah, Ansar A. Pathan, Fatimah S. Alhamlan, Emmanouil Karteris, Uday Kishore

**Affiliations:** ^1^Biosciences, College of Health and Life Sciences, Brunel University London, Uxbridge, UK; ^2^Department of infection and Immunity, King Faisal Specialist Hospital and Research Centre, Riyadh, Saudi Arabia; ^3^Institute of Environment, Heath and Societies, Brunel University London, Uxbridge, UK

**Keywords:** complement, C1q, ovarian cancer, apoptosis, TNF, mTOR

## Abstract

Complement protein C1q is the first recognition subcomponent of the complement classical pathway that plays a vital role in the clearance of immune complexes, pathogens, and apoptotic cells. C1q also has a homeostatic role involving immune and non-immune cells; these functions not necessarily involve complement activation. Recently, C1q has been shown to be expressed locally in the microenvironment of a range of human malignant tumors, where it can promote cancer cell adhesion, migration, and proliferation, without involving complement activation. C1q has been shown to be present in the ascitic fluid formed during ovarian cancers. In this study, we have examined the effects of human C1q and its globular domain on an ovarian cancer cell line, SKOV3. We show that C1q and the recombinant globular head modules induce apoptosis in SKOV3 cells in a time-dependent manner. C1q expression was not detectable in the SKOV3 cells. Exogenous treatment with C1q and globular head modules at the concentration of 10 µg/ml induced apoptosis in approximately 55% cells, as revealed by immunofluorescence microscopy and FACS. The qPCR and caspase analysis suggested that C1q and globular head modules activated tumor necrosis factor (TNF)-α and upregulated Fas. The genes of mammalian target of rapamycin (mTOR), RICTOR, and RAPTOR survival pathways, which are often overexpressed in majority of the cancers, were significantly downregulated within few hours of the treatment of SKOV3 cells with C1q and globular head modules. In conclusion, C1q, *via* its globular domain, induced apoptosis in an ovarian cancer cell line SKOV3 *via* TNF-α induced apoptosis pathway involving upregulation of Bax and Fas. This study highlights a potentially protective role of C1q in certain cancers.

## Introduction

C1q is the first subcomponent of the C1 complex that recognizes the IgG- or IgM-containing immune complexes and initiates the complement classical pathway. It can bind to various self- and non-self ligands and bring about a range of homeostatic functions including clearance of pathogens and apoptotic cells (1). Human C1q molecule is composed of 18 polypeptide chains (6A, 6B, and 6C). Each C1q chain has a short N-terminal region, a triple-helical collagen region, and a C-terminal globular (gC1q) domain (1, 2). Recently, a wide range of immunomodulatory functions of C1q have become evident that are independent of its involvement in the complement activation (3, 4); these include modulation of dendritic cell functions (5), cancer progression (6), and neuronal synapse pruning (7).

Ovarian cancer is the sixth most frequently diagnosed cancer among women worldwide and has the highest mortality rate than any other female reproductive system-associated cancer. Approximately 70% of the ovarian cancers are diagnosed at an advanced stage III or IV, with nearly 85% expected mortality (8). The intraperitoneally ascitic fluid (AF) formed during ovarian cancer has been shown to have high levels of complement components such as C3a and soluble C5b–9, which form the membrane attack complex, suggesting potential for the activation of complement *in vivo*. However, complement activation appears to be dampened due to the expression of membrane regulators such as CD46, CD55, and CD59 on the ovarian cancer cells, rendering complement system as an inefficient immune surveillance mechanism. The malignant ovarian tumor cells isolated from AF have been shown to have C1q and C2 deposited on the surface, which were rendered susceptible to complement-mediated killing by AF in the presence of anti-CD59-neutralizing antibody (9). In a recent study, the presence of C1q has been shown in the stroma and vascular endothelium of a number of human malignant tumors, including lung adenocarcinoma, melanoma, colon adenocarcinoma, breast adenocarcinoma, and pancreatic carcinoma (6). This has raised a tumor growth-fostering role for locally synthesized C1q *via* promotion of adhesion, migration, and proliferation.

The importance of complement in cancer immunotherapy has acquired great interest recently. A broad array of cell surface tumor-associated antigens that are overexpressed, mutated, or partially expressed, as compared to normal tissues, have offered various antibody targets in different cancers (10). A number of these anti-cancer antibodies work *via* receptor or checkpoint blockade or as an agonist, apoptosis induction, immune-mediated cytotoxicity either *via* complement or antibody, and T cell function regulation. In addition, therapeutic antibodies targeting growth factors and their receptors such as epidermal growth factor receptor, insulin-like growth factor 1 receptor, tumor necrosis factor (TNF)-related apoptosis-inducing ligand receptors, and receptor activator nuclear factor-κB ligand (RANKL) have also been exploited for cancer treatment (11).

In this study, we sought to investigate the complement-independent effects of exogenous C1q and recombinant forms of globular head modules on an ovarian cancer cell line, SKOV3.

## Materials and Methods

### Cell Culture and Treatments

A human ovarian clear cell adenocarcinoma cell line, SKOV3 (ATCC, Rockville, MD, USA) was used as an *in vitro* model for epithelial ovarian cancer. Cells were cultured in DMEM-F12 media containing 10% v/v fetal calf serum, 2mM l-glutamine, and penicillin (100 U/ml)/streptomycin (100 µg/ml) (Thermo Fisher). Cells were grown at 37°C under 5% v/v CO_2_ until 80–90% confluency was reached.

### Purification of Human C1q

Human C1q was purified as published earlier (12). Briefly, freshly thawed human plasma was made 5 mM EDTA, centrifuged at 5,000 × *g* for 10 min, and any aggregated lipids were removed using Whatmann filter paper (GE Healthcare, UK). The plasma was then incubated with non-immune IgG-Sepharose (GE Healthcare, UK) for 2 h at room temperature. C1q bound IgG-Sepharose was washed extensively with 10 mM HEPES, 140 mM NaCl, 0.5 mM EDTA, and pH 7.0 before eluting C1q with CAPS (*N*-cyclohexyl-3-aminopropanesulfonic acid) buffer (100 mM CAPS, 1 M NaCl, 0.5 mM EDTA, pH 11). The eluted C1q fractions were then passed through a HiTrap Protein G column (GE Healthcare, UK) to remove IgG contaminants, followed by dialysis against 0.1 M HEPES buffer, pH 7.5.

### Recombinant Expression and Purification of ghA, ghB, and ghC Modules of Human C1q

The recombinant forms of the globular head regions of human C1q A (ghA), B (ghB), and C (ghC) chains were expressed as fusions to *Escherichia coli* maltose-binding protein (MBP) and purified, as reported previously (13). Expression constructs pKBM-A, pKBM-B, and pKBM-C were transformed into *E. coli* BL21 (Invitrogen) cells in the presence of ampicillin (100 µg/ml). The primary bacterial culture was grown overnight by inoculating a single colony in 25 ml of Luria-Bertani medium containing ampicillin. The bacterial culture was then grown in a 1 L batch until OD_600_ 0.6 and then induced with 0.4 mM isopropyl β-d-thiogalactoside (IPTG) (Sigma-Aldrich, UK) for 3 h at 37°C on a shaker and centrifuged (5,000 × *g*, 4°C, 15 min). Subsequently, the cell pellet for each fusion protein was lysed in 50 ml lysis buffer (20 mM Tris–HCl, pH 8.0, 0.5 M NaCl, 0.2% v/v Tween 20, 1 mM EGTA pH 7.5, 1 mM EDTA pH 7.5, and 5% v/v glycerol) containing lysozyme (100 µg/ml, Sigma-Aldrich, UK) and 0.1 mM phenylmethylsulfonyl fluoride (PMSF; Sigma-Aldrich, UK) at 4°C for 30 min. The resultant cell suspension was sonicated at 60 Hz for 30 s with an interval of 2 min each (12 cycles) and centrifuged (16,000 × *g* for 30 min). The supernatant was diluted 5-fold with buffer I (20 mM Tris–HCl, pH 8.0, 100 mM NaCl, 0.2% v/v Tween 20, 1 mM EDTA pH 7.5, and 5% v/v glycerol) and passed through an amylose resin column (50 ml; New England Biolabs). The column was then washed extensively with buffer I (150 ml), followed by buffer II (250 ml of buffer I without Tween 20) before eluting 1 ml fractions of fusion proteins with 100 ml buffer II containing 10 mM maltose. The peak fractions were then passed through Pierce™ High Capacity Endotoxin Removal Resin (Qiagen) to remove lipopolysaccharide. Endotoxin levels in the purified protein samples were analyzed using the QCL-1000 Limulus amebocyte lysate system (Lonza). The assay was linear over a range of 0.1–1.0 EU/ml (10 EU = 1 ng of endotoxin), and the amount of endotoxin levels was <4 pg/μg of the recombinant proteins.

### Fluorescence Microscopy

SKOV3 cells (0.5 × 10^5^) were grown on coverslips and incubated with human C1q, ghA, ghB, or ghC (10 µg/ml) in a serum-free DMEM-F12 medium for 1 h for analyzing cell binding, 24 h for apoptosis induction, and 15 h for mammalian target of rapamycin (mTOR) activation analysis. For binding analysis, the coverslips were washed three times with PBS and then incubated with rabbit anti-human C1q polyclonal antibody (MRC Immunochemistry Unit, Oxford, 1:200) for C1q and rabbit anti-MBP polyclonal antibody (Thermo Fisher) for MBP fusions of the globular head modules. Coverslips were washed three times with PBS, and then incubated with Alexa Fluor^®^ 488 (1:500, Thermo Fisher) and Hoechst (1:10,000, Thermo Fisher) for immunofluorescence analysis. Apoptosis was analyzed *via* immunofluorescence using a FITC-Annexin V apoptosis detection kit with propidium iodide (PI) (Biolegend). After 24 h incubation with proteins, the coverslips were then incubated with Annexin V-binding buffer containing FITC Annexin V (1:200) and PI (1:200) for 15 min and washed twice with PBS before mounting on the slides to visualize under a HF14 Leica DM4000 microscope. For mTOR analysis, following the 15-h incubation with proteins, the coverslips were washed three times with PBS. The cells were fixed and permeabilized using ice-cold 100% methanol at −20°C for 10 min, followed by 1 h incubation with rabbit anti-human mTOR (1:500, Sigma), and then 1 h incubation with Alexa Fluor^®^ 488 (rabbit anti-human 1:500, Thermo Fisher) and Hoechst (1:10,000, Thermo Fisher) for immunofluorescence analysis. In order to detect caspase 3 activation, the protein-treated SKOV3 cells were fixed and permeabilized using ice-cold 100% methanol at −20°C for 10 min, followed by 1 h incubation with rabbit anti-human cleaved caspase 3 (1:500, Cell Signaling), and then 1 h incubation with secondary antibody (anti-rabbit) probed with CY3 (1:500, Thermo Fisher) and Hoechst (1:10,000, Thermo Fisher) for immunofluorescence analysis.

### Dye Exclusion Assay

SKOV3 cells (0.1 × 10^6^) were grown in a 12-well plate and incubated with C1q, ghA, ghB, ghC, or MBP (10 µg/ml) along with an untreated control in serum-free DMEM-F12 for 24 h. Cells were detached using 5 mM EDTA, pH 8.0 and centrifuged at 1,200 × *g* for 5 min. The cell pellet was re-suspended in 1 ml DMEM-F12, and 10 µl of cell suspension was stained with 10 µl of Trypan blue (60%; Sigma-Aldrich, UK) for cell counting using hemocytometer. The viable cells, i.e., unstained cells, were counted in 5 different optical fields with a threshold value of 200 cells per field.

### MTT Assay

MTT [3-(4,5-dimethylthiazol-2-yl)-2,5-diphenyltetrazolium bromide] (Thermo Fisher) assay was performed by incubating SKOV3 cells (0.1 × 10^5^) in a 96-well microtiter plate with C1q, ghA, ghB, ghC, or MBP (10 µg/ml each) and an untreated control in serum-free DMEM-F12 medium for 24 h, followed by incubation with 50 µg/µl MTT (5 mg/ml stock) per well for 4 h at 37°C. Majority of the media was removed leaving behind 25 µl per well, which was mixed thoroughly with 50 µl of dimethyl sulfoxide (DMSO) and incubated for another 10 min at 37°C. The absorbance was read at 570 nm using a plate reader.

### Flow Cytometry

SKOV3 cells (0.1 × 10^7^) were incubated with C1q, ghA, ghB, or ghC (10 µg/ml), along with an untreated control, in a six-well plate for 24 h, followed by cell detachment using 5 mM EDTA and centrifugation at 1,200 × g for 5 min. FITC Annexin V apoptosis detection kit with PI (Biolegend) was used, as per the manufacturer’s instructions. Compensation parameters were acquired using unstained, untreated FITC stained, and untreated PI stained SKOV3 cells. SKOV3 cells (0.1 × 10^4^) were acquired for each experiment and compensated before plotting the acquired data. For protein-binding analysis, SKOV3 cells (0.5 × 10^6^) were incubated with C1q, ghA, ghB, and ghC (10 µg/ml) and BSA-treated cells as a negative control. Cells were incubated at 4°C for 1 h, followed by 1 h incubation with rabbit anti-human C1q polyclonal antibody (1:200) for C1q and rabbit anti-MBP (Thermo Fisher, 1:200) for globular head modules treated cells as well as their respective BSA-treated controls. The cells were then incubated with Alexa Fluor^®^ 488 (1:1,000, Thermo Fisher) for 1 h and cells were washed with PBS three times between each step. Compensation parameters were acquired using unstained, untreated Alexa Fluor^®^ 488-stained SKOV3 cells. SKOV3 cells (0.1 × 10^4^) were acquired using Novocyte Flow Cytometer for each experiment and compensated before plotting the acquired data.

### Western Blot

SKOV3 cells (0.1 × 10^7^) were plated in a six-well plate (Nunc) and incubated with C1q, ghA, ghB, or ghC (10 µg/ml), along with an untreated control in serum-free DMEM-F12 medium for 12 and 24 h. The cells were lysed within the wells using lysis buffer (50 mM Tris–HCL, pH 7.5, 10% v/v glycerol, and 150 mM NaCl) over ice for 10 min, before being gently transferred to a pre-cooled microcentrifuge tube, and centrifuged for 15 min at 13,000 × *g* at 4°C. The cell lysate was mixed with the treatment buffer (50 mM Tris pH 6.8, 2% β-merceptoethanol, 2% SDS, 0.1% bromophenol blue, and 10% glycerol) in order to run on SDS-PAGE (12% w/v) for 90 min at 120 V. The SDS-PAGE separated proteins were then electrophoretically transferred onto a nitrocellulose membrane (Sigma) using transfer buffer (25 mM Tris, 190 mM glycine, and 20% methanol, pH 8.3) for 2 h at 320 mA, followed by blocking with 5% w/v dried milk powder (Sigma) in 100-ml PBS at room temperature for 2 h on a rotatory shaker. The membrane was washed with PBST (PBS + 0.05% Tween 20) three times, 10 min each, and incubated with primary anti-human C1q (polyclonal antibody), anti-caspase 9, anti-caspase 3, or anti-cleaved caspase 3 antibodies (1:1000 Cell Signaling Technology) at 4°C overnight. The membrane was washed with PBST (three times, 10 min each) before incubating with secondary anti-rabbit IgG HRP-conjugate (1:1,000; Promega) for 1 h at room temperature. The bands were visualized using enhanced chemiluminescence (Thermo Fisher) in the Biorad ChemiDoc MP imaging system, or using 3,3-diaminobenzidine (DAB) substrate kit (Thermo Fisher).

### Quantitative RT-PCR

SKOV3 cells (0.5 × 10^6^) were incubated with C1q, ghA, ghB, or ghC (10 µg/ml) for various time points, and the centrifuged cell pellets were stored at −80°C. GenElute Mammalian Total RNA Purification Kit (Sigma-Aldrich, UK) was used, as per the manufacturer’s instructions, to extract total RNA, followed by treatment with DNase I (Sigma-Aldrich, UK) to remove any DNA contaminants. The concentration and purity of total RNA were determined by measuring the absorbance at 260 nm and 260:280 nm ratio, respectively, using NanoDrop 2000/2000c (Thermo Fisher Scientific). Total RNA (2 µg) was used to synthesize cDNA using High Capacity RNA to cDNA Kit (Applied Biosystems).

Forward and reverse primer sequences (Table 1) were designed using the web-based Basic Local Alignment Search Tool and Primer-BLAST (http://blast.ncbi.nlm.nih.gov/Blast.cgi). The qPCR reactions were performed to measure the mRNA expression level of various target genes. Each reaction was conducted in triplicates and consisted of 5 µl Power SYBR Green MasterMix (Applied Biosystems), 75 nM of forward and reverse primers, and 500 ng cDNA, making up to a 10 µl final volume per well. Relative mRNA expression was determined by qPCR using the 7900HT Fast Real-Time PCR System (Applied Biosystems). Samples were initially incubated at 50°C and 95°C for 2 and 10 min, respectively, followed by amplification of the template for 40 cycles (each cycle involved 15 s at 95°C and 1 min at 60°C). The gene expression was normalized using the expression of human 18S rRNA as an endogenous control. The cycle threshold (Ct) mean value for each target gene was used to calculate the relative expression using the relative quantification (RQ) value and formula: RQ = 2^−ΔΔCt^, which was compared with the relative expression of 18S.

**Table 1:** **Target genes and terminal primers used in the qPCR analysis**.

Target gene	Forward primer	Reverse primer
18S	5′-ATGGCCGTTCTTAGTTGGTG-3′	5′-CGCTGAGCCAGTCAGTGTAG-3′
Bax	5′-TGCTTCAGGGTTTCATCCAGG-3′	5′-GGAAAAAGACCTCTCGGGGG-3′
Fas	5′-ACACTCACCAGCAACACCAA-3′	5′-TGCCACTGTTTCAGGATTTAA-3′
Mammalian target of rapamycin (mTOR)	5′-TGCCAACTATCTTCGGAACC-3′	5′-GCTCGCTTCACCTCAAATTC-3′
RICTOR	5′-GGAAGCCTGTTGATGGTGAT-3′	5′-GGCAGCCTGTTTTATGGTGT-3′
RAPTOR	5′-ACTGATGGAGTCCGAAATGC-3′	5′-TCATCCGATCCTTCATCCTC-3′
Tumor necrosis factor (TNF)-α	5′-GTATCGCCAGGAATTGTTGC-3′	5′-AGCCCATGTTGTAGCAAACC-3′
NF-κB	5′-TGAGGTACAGGCCCTCTGAT-3′	5′-GTATTTCAACCACAGATGGCACT-3′

### Statistical Analysis

GraphPad Prism 6.0 was used to make graphs, and the statistical analysis was carried out using an unpaired one-way ANOVA test. Significant values were considered based on *p < 0.05, *p < 0.01, and ***p < 0.001 between treated and untreated conditions. Error bars show the SD or SEM, as indicated in the figure legends.

## Results

### Human C1q and Recombinant Globular Head Modules Bind SKOV3

**Figure 1:**
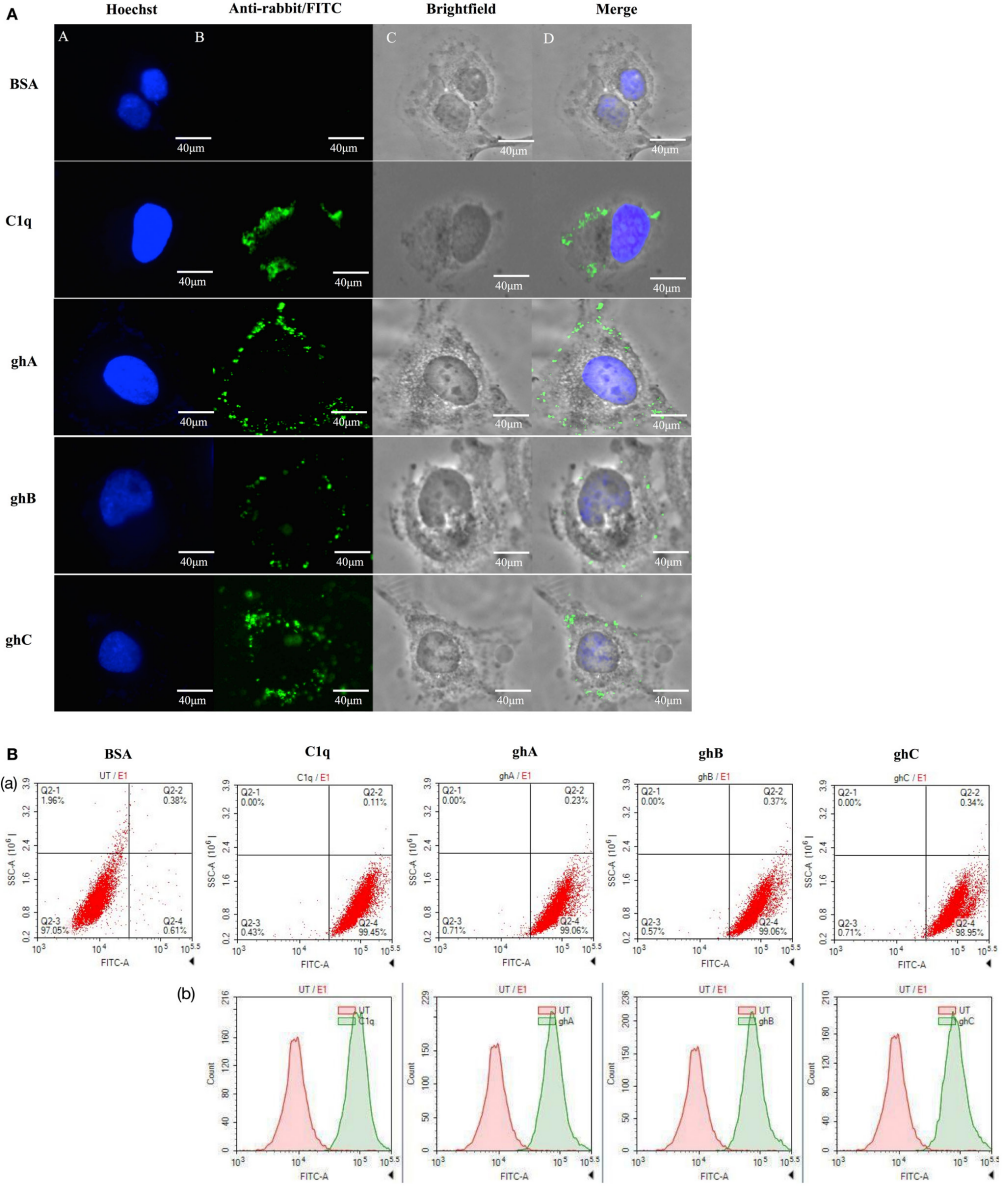
**(A)** Binding of human C1q and recombinant globular head modules, ghA, ghB, and ghC (10 µg/ml; 1 h incubation) to SKOV3 cells using immunofluorescence microscopy. Panel A shows the nucleus of the cells stained with Hoechst. Panel B shows the cells probed with anti-C1q (C1q) and anti-maltose-binding protein (MBP) (globular heads) polyclonal antibodies, followed by anti-rabbit IgG labeled with FITC; the bound proteins are visible on the cell membrane (panels C and D). **(B)** Flow cytometric analysis to show binding of human C1q and ghA, ghB, and ghC (10 µg/ml) to SKOV3 cells after 1 h incubation. Panel a shows the number of cells probed with anti-C1q (C1q) and anti-MBP (globular heads) antibodies followed by anti-rabbit IgG labeled with FITC, as compared to the untreated cells. Panel b shows the shift in the fluorescent intensity from untreated to treated cells.

The qualitative binding analysis of C1q and recombinant form of individual globular head (ghA, ghB, and ghC) modules (10 µg/ml) to SKOV3 cells using immunofluorescence microscopy revealed the membrane localization of these proteins following 1 h incubation at 4°C (Figure 1A). The nucleus, stained with Hoechst, showed a blue fluorescence (Figure 1A, panel A). The bound proteins localized on the cell membrane *via* green fluorescence; all the four proteins showed similar binding pattern (Figure 1A, panels B and D). FACS analysis revealed similar binding pattern, consistent with the observations made under the immunofluorescence microscopy. SKOV3 cells, treated with C1q and globular head modules, were over 90% positive in the FITC positive quadrant, as compared to the control protein, BSA (Figure 1B).

### C1q and Recombinant Globular Head Modules Reduce Cellular Viability *via* Induction of Apoptosis in SKOV3 Cells

**Figure 2:**
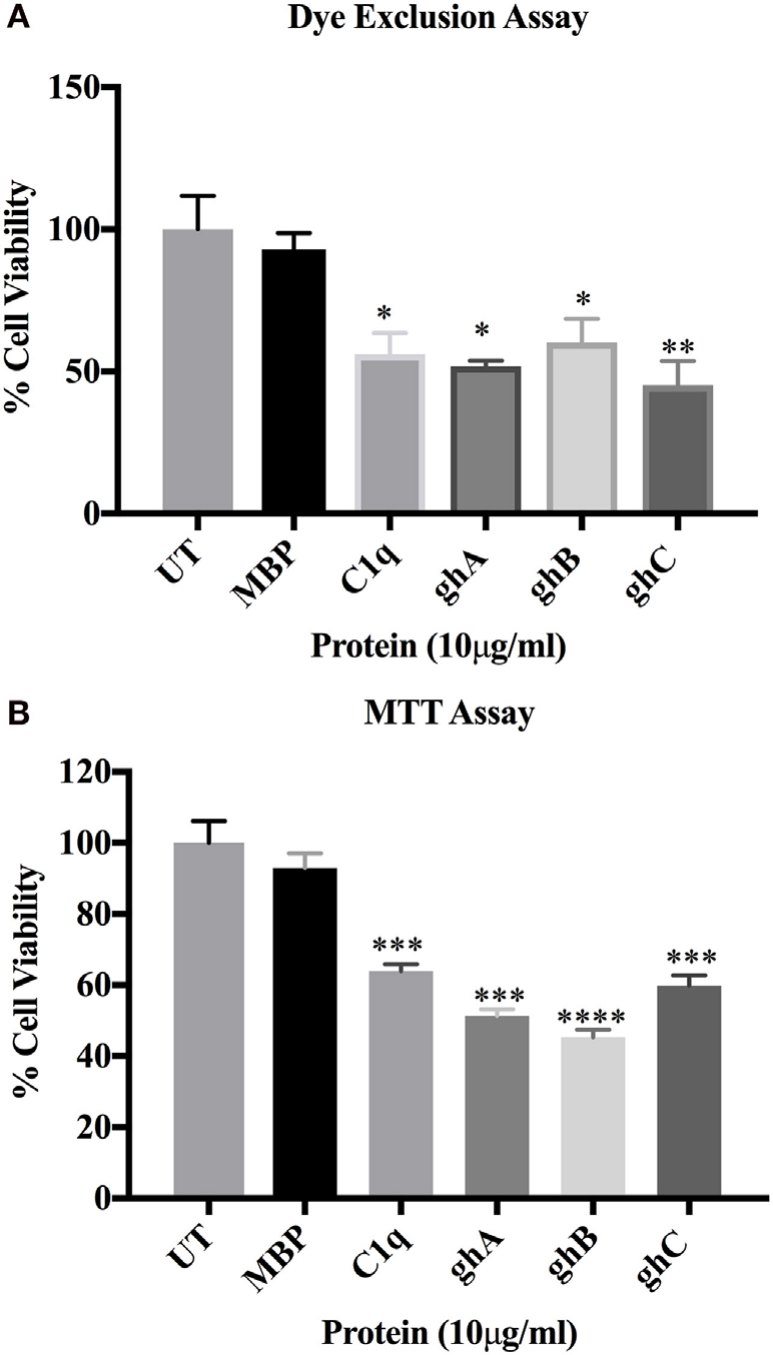
**SKOV3 cell viability assay ***via*** Trypan blue exclusion (A) and MTT (B) following treatment with human C1q, ghA, ghB, ghC, and maltose-binding protein (MBP) (10 μg/ml)and untreated control for 24 h (±SEM, of three independent experiments)**. Cell numbers were reduced by approximately 50% in the treated as compared to untreated and MBP controls. Significance was established using the unpaired one-way ANOVA test (*p < 0.05, *p < 0.01, and ***p < 0.001) (*n* = 6).

**Figure 3:**
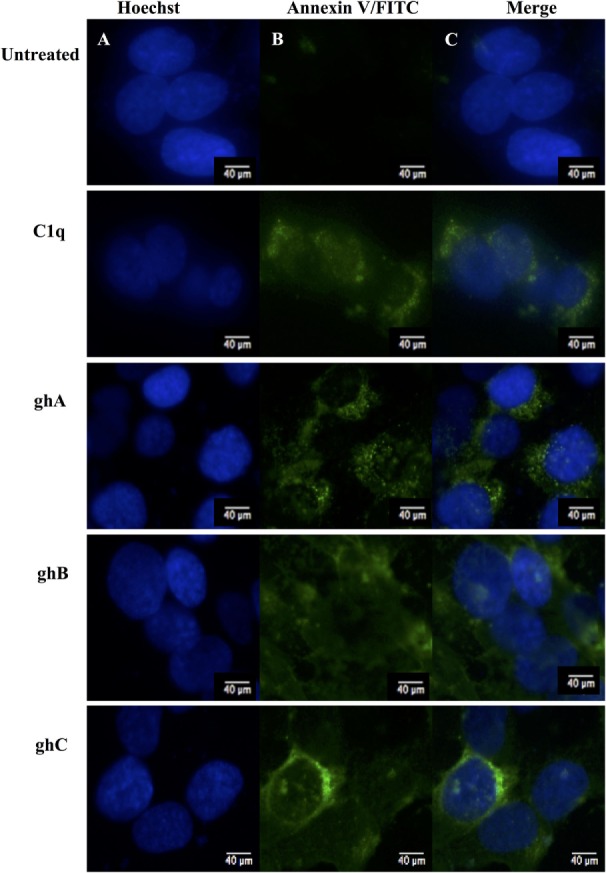
**Analysis of apoptosis using immunofluorescence microscopy in SKOV3 cells treated with human C1q, ghA, ghB, and ghC (10 µg/ml) and an untreated control after 24 h**. Panel A shows the nucleus stained with Hoechst. Panel B shows the cell membrane integrity marker FITC Annexin V, which binds to PS of the cell membrane of cells undergoing apoptosis. No FITC was detected in the untreated SKOV3 cells.

C1q and globular head modules (at 10 µg/ml concentration) were incubated with SKOV3 cells for 24 h. Trypan blue dye exclusion (Figure 2A) revealed a significant decrease in the cell viability following incubation with C1q (~50%), ghA (~50%), ghB (~40%), and ghC (~55%). MTT assay also confirmed the above mentioned observations (Figure 2B). Furthermore, the qualitative apoptosis analysis was performed using immunofluorescence microscopy. A significant proportion of SKOV3 cells, when treated with C1q proteins (10 µg/ml) for 24 h, stained positive for cell membrane integrity marker, Annexin V (conjugated to FITC), which binds to phosphatidylserine (PS) of the membrane of cells undergoing apoptosis (Figure 3, panel B). The nucleus was stained using Hoechst, which showed blue fluorescence (Figure 3, panel A). No FITC fluorescence was detected in the untreated cells, suggesting that the integrity of the cell membrane was intact, and hence, the cells were still viable (Figure 3).

**Figure 4:**
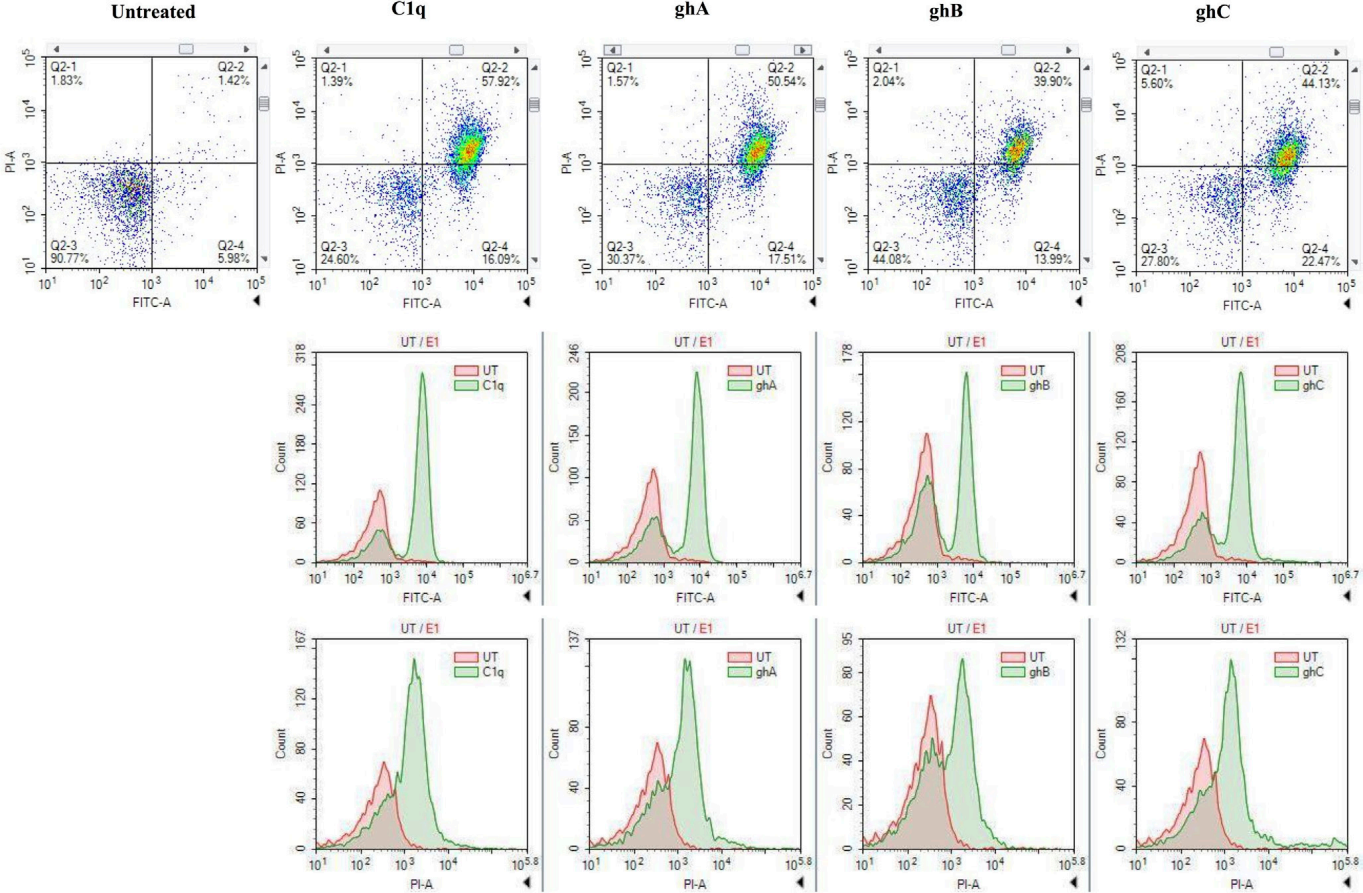
**The quantitative analysis of apoptosis using FACS**. SKOV3 cells, treated with C1q and globular head modules for 24 h, yielded approximately 50% (C1q, ghA, ghB, and ghC quadrant Q2-2) cells positive for both FITC and PI, significantly higher than 1.42% untreated control cells (untreated, quadrant Q2-2). Approximately 20% cells stained positive for FITC only (C1q, ghA, ghB, and ghC quadrant Q2-4).

FACS analysis revealed that the treatment of SKOV3 cells with C1q (10 µg/ml) for 24 h yielded approximately 58% (C1q, quadrant Q2-2; Figure 4) cells positive for both FITC (Annexin V marker) and PI (stains DNA) quadrant, significantly higher than 1.42% untreated control cells (UT, quadrant Q2-2; Figure 4), which represented a cell population undergoing late apoptosis. In addition, 16% of cells stained positive for FITC only (C1q, quadrant Q2-4; Figure 4), which represented an early apoptotic cell population.

Similar trends were also observed when SKOV3 cells were treated with ghA, ghB, and ghC; approximately 50% (ghA and ghC) and 40% (ghB) of cells had undergone apoptosis after 24 h treatment as seen in quadrant Q2-2 (ghA, ghB, and ghC in Figure 4). Approximately, 15–20% of early apoptotic cells stained positive for FITC alone (quadrant Q2-4; Figure 4) following treatment with each globular head module. In the untreated cells, approximately 90% of the cell population was both FITC and PI negative (UT, quadrant Q2-3; Figure 4). A shift in the florescence intensity in the treated SKOV3 cells, as compared to the untreated cells, further confirmed these observations (Figure 4).

### C1q and Individual Globular Head Modules Upregulate TNF-α Transcripts by SKOV3 Cells with Concomitant Upregulation of NF-κB

**Figure 5:**
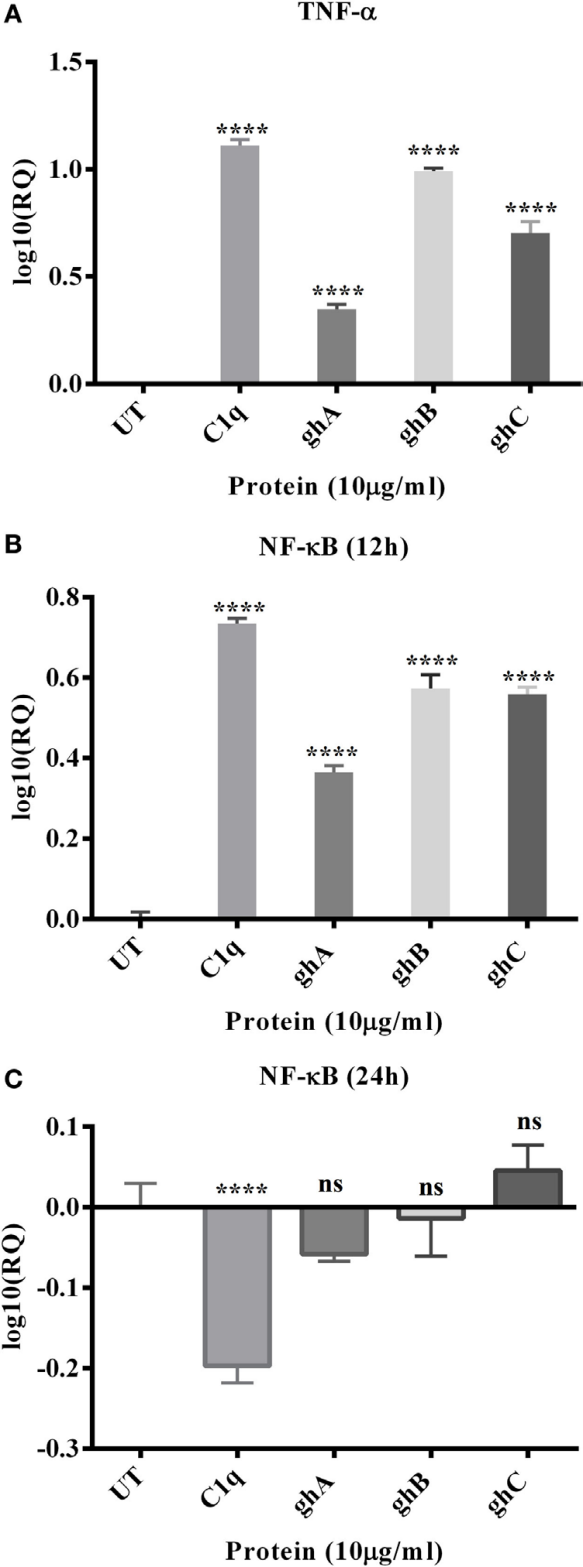
**Relative quantification comparisons of TNF-α (A) and NF-κB (B,C) mRNA expression in SKOV3 cells treated with C1q, ghA, ghB, and ghC**. The transcriptional expressions of both TNF-α and NF-κB were upregulated (log_10_ 0.5- to 1-fold) after 12 h treatment and NF-κB at 24 h was downregulated. Significance was ascertained using the unpaired one-way ANOVA test (*p < 0.05, *p < 0.01, and ***p < 0.001) (*n* = 3).

The mRNA expression level of pro-inflammatory cytokine, TNF-α, was significantly upregulated at 12 h following C1q (log_10_ 1.2-fold) and globular head modules; ghA (log_10_ 0.4-fold), ghB (log_10_ 1-fold), and ghC (log_10_ 0.7-fold) treatment in comparison to an untreated control, as determined by qPCR (Figure 5A). NF-κB was also significantly upregulated (log_10_ 0.7-fold) at 12 h, as anticipated, consistent with TNF-α upregulation (Figure 5B). Both TNF-α (not shown) and NF-κB mRNA expressions were downregulated by 24 h (Figure 5C), which could be attributed to the late apoptosis stage. In addition, TNF-α transcriptional upregulation occurred at an earlier time point for ghA, i.e., at 6 h as compared to the other treated samples (data not shown). These results prompted us to further investigate the mRNA expression of pro-apoptotic makers of intrinsic (Bax) and extrinsic (Fas) apoptosis pathways.

### C1q Upregulates Expression of Pro-apoptotic Bax and Fas in SKOV3 Cells

**Figure 6:**
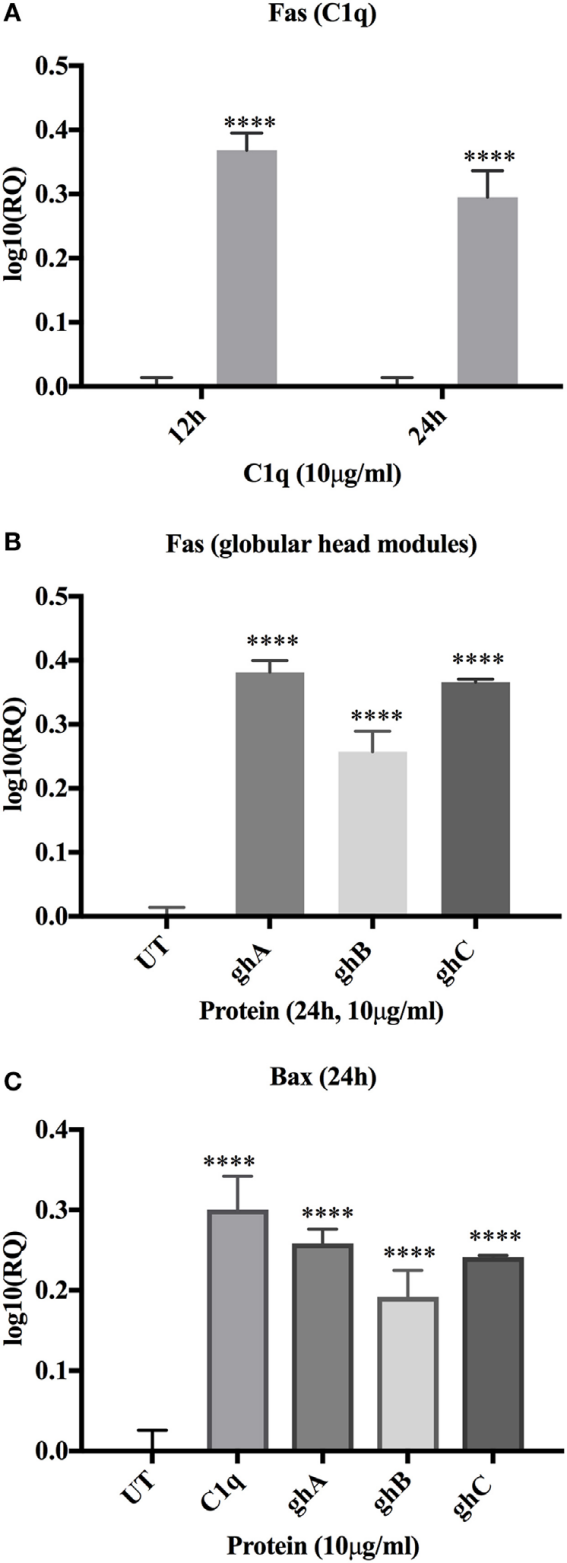
**Relative quantification comparisons of Fas (A) and Bax (B,C) mRNA expression in SKOV3 cells treated with C1q, ghA, ghB, and ghC (10 µg/ml)**. Fas expression was upregulated (~log_10_ 0.5-fold) at 12 h (C1q only) and 24 h (both for C1q and globular head modules). Bax expression was upregulated (~log_10_ 0.5-fold) at 24 h. Significance was determined using the unpaired one-way ANOVA test (*p < 0.05, *p < 0.01, and ***p < 0.001) (*n* = 3).

C1q- and globular head module-mediated apoptosis was further investigated by analyzing the expression of pro-apoptotic genes, Bax and Fas, *via* qPCR. Fas was transcriptionally upregulated in the C1q (log_10_ 0.4-fold) treated SKOV3 cells, most significantly at 12 and 24 h relative to the untreated control cells (Figure 6A). The globular head module-treated cells showed minimal mRNA expression levels at 12 h (data not shown) and significant upregulation of Fas at 24 h (log_10_ 0.4-fold) (Figure 6B). Similar trend of minimal mRNA expression at 12 h (data not shown) and significant upregulation at 24 h (Figure 6C) were observed for Bax mRNA expression in case of ghA, ghB, or ghC. The activation of apoptotic pathway causes the loss of cell membrane integrity, which was previously observed in the immunofluorescence microscopy at 24 h, consistent with the upregulation of mRNA expression of these genes. To further validate these results, the activation of Caspase 3 was investigated *via* western blot.

### C1q and the Globular Head Modules Activate Caspase 3 in SKOV3 Cells

**Figure 7:**
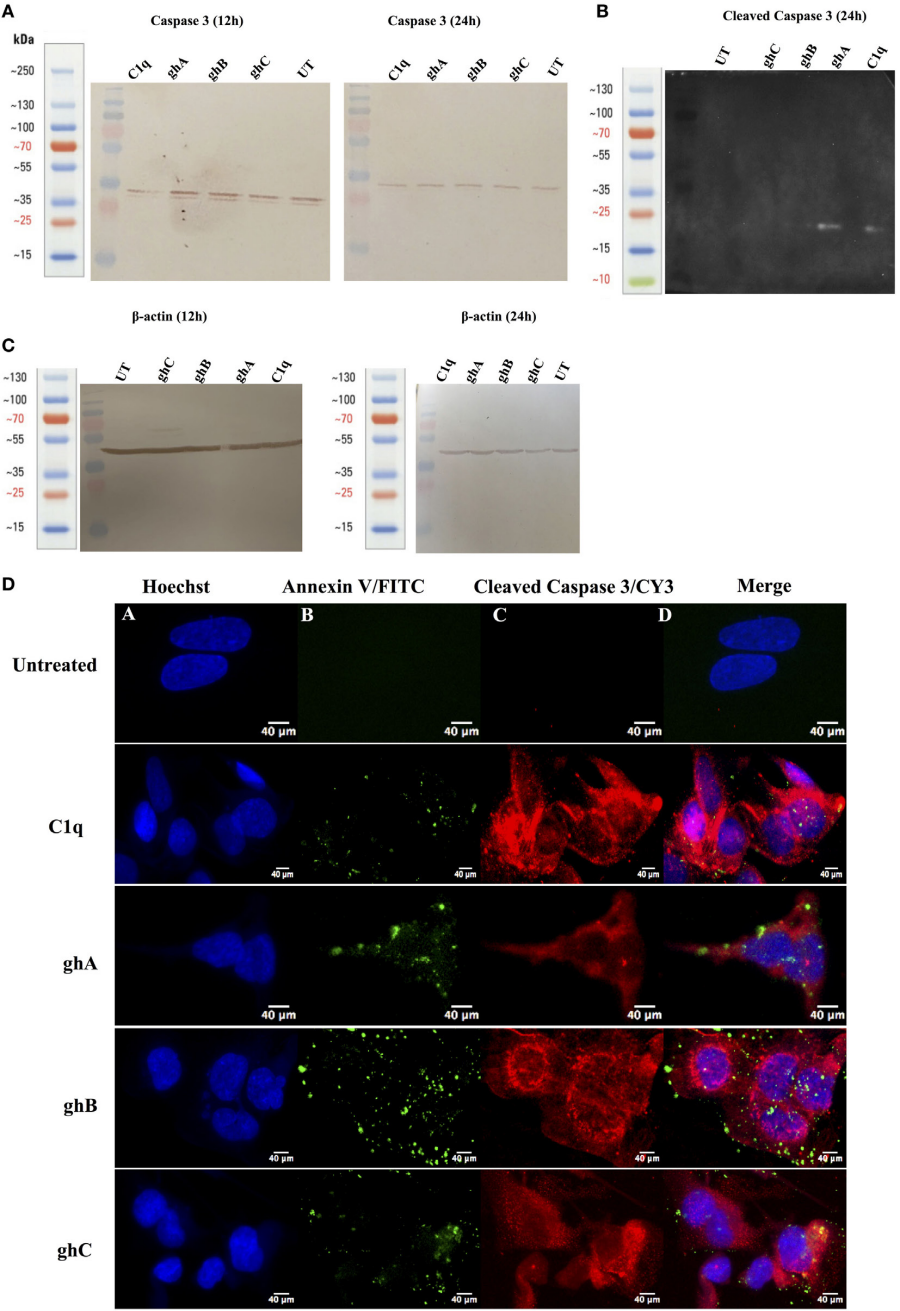
**Caspase activation in SKOV3 cells following treatment with C1q and globular head modules**. **(A)** Western blot analysis of full-length/total caspase 3 at 32 kDa after 12 and 24 h of the treatment with C1q, ghA, ghB, ghC, and untreated. **(B)** The cleaved caspase 3 was observed at 17 kDa after 24 h of treatment with C1q, ghA, ghB, ghC, and untreated **(C)** Western blot analysis for β-actin as a loading control for 12 h and 24 h at 45 kDa. **(D)** The activation of caspase 3 was also shown by immunofluorescence microscopy at 24 h in parallel with apoptosis staining for Annexin V-FITC, where activated caspase 3 was clearly visible in the cytoplasm probed with CY3 at 24 h.

Apoptotic markers, caspase 3 and cleaved caspase 3, were detected in the SKOV3 cells, treated with C1q and globular head modules, in comparison with the untreated cells. The cleaved caspase 3 was detected at 17 kDa after 24 h of C1q, ghA, and ghB treatment in the western blot (Figure 7B). A reduction in the abundance of the full length at 32 kDa was seen after 24 h in comparison to 12 h (Figure 7A) β-actin (45 kDa) was used as a loading control for 12 h and 24 h (Figure 7C). In addition, the activation of caspase 3 was also seen by immunofluorescence in parallel to apoptosis staining for Annexin V-FITC at 24 h following treatment with C1q or globular head modules. Activated caspase 3 was clearly visible in the cytoplasm probed with Cy3, suggesting that the SKOV3 cells were in the early stages of apoptosis as the membrane was stained positively for Annexin V-FITC, in comparison to the control where no staining was detected (Figure 7D). The cleaved caspase 3 in the ghC treatment sample could not be detected *via* western blot; however, activated caspase 3 was detected *via* immunofluorescence.

### C1q, ghA, ghB, and ghC Downregulate Prosurvival Factors Such as RICTOR, mTOR, and RAPTOR

**Figure 8:**
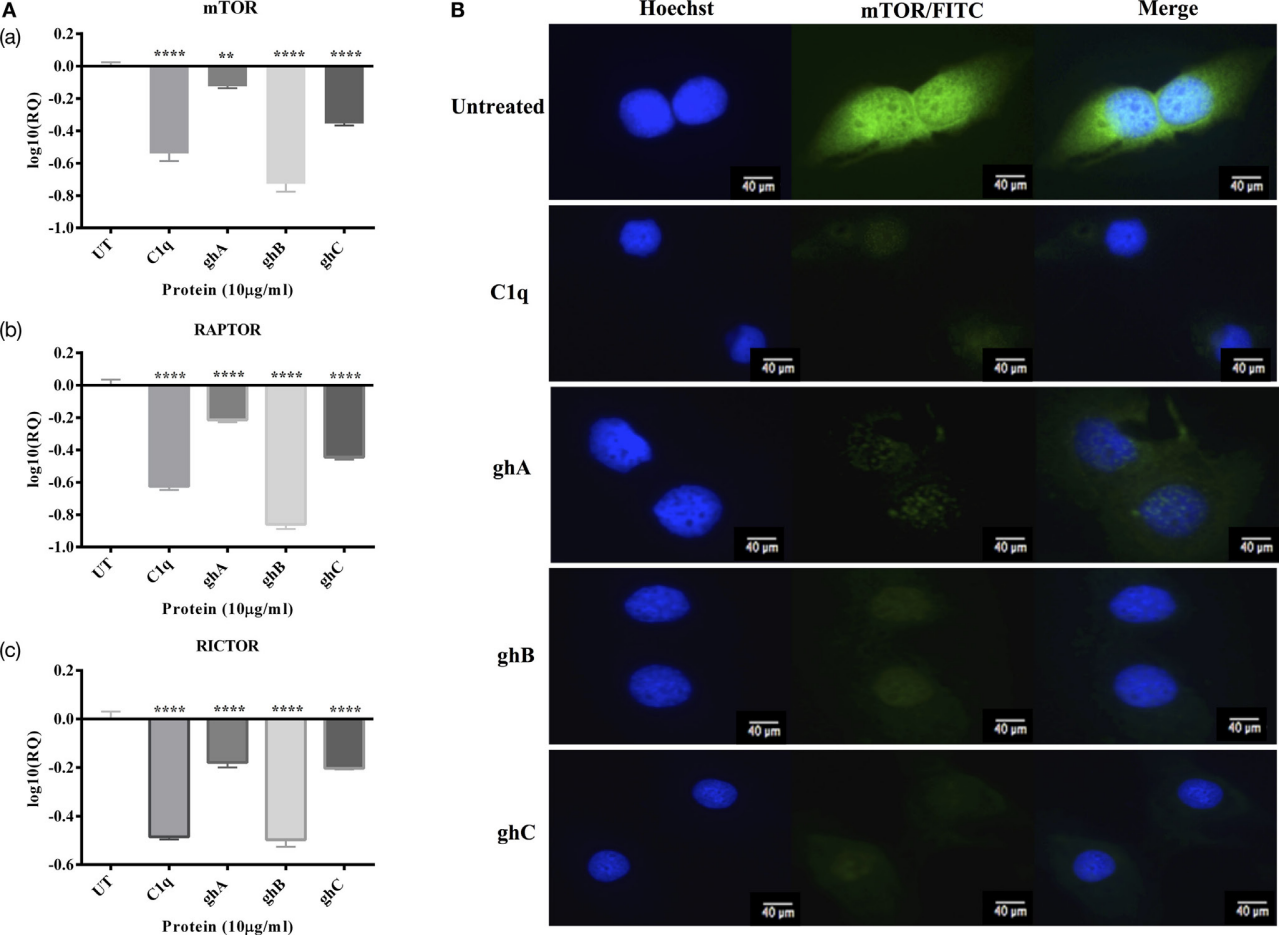
**(A) Relative quantification comparisons of mammalian target of rapamycin (mTOR) (a), RAPTOR (b), and RICTOR (c) mRNA expression in SKOV3 cells treated with C1q, ghA, ghB, and ghC (10 µg/ml) at 6 h**. Significance was obtained using the unpaired one-way ANOVA test (*p < 0.05, *p < 0.01, and ***p < 0.001) (*n* = 3). **(B)** Immunofluorescence microscopy to show the presence of mTOR detected abundantly in the cytoplasm (green) of the untreated cells, compared to SKOV3 cells treated with C1q and globular heads at 15 h time point.

In addition to the apoptosis markers, we also investigated the expression of mTOR signaling pathways since it is frequently detected in ovarian cancers (14). The mRNA expressions of mTOR, RICTOR, and RAPTOR (Figure 8A) were significantly downregulated (~log_10_ 0.5-fold) at 6 h, thereby suggesting that the effects of C1q and globular head modules can compromise mTOR signaling within the first few hours of the treatment. Additionally, the presence of mTOR was detected abundantly in the cytoplasm (green) of the untreated cells, compared to SKOV3 cells treated with C1q and globular heads at 15 h time point *via* immunofluorescence microscopy (Figure 8B).

## Discussion

**Figure 9:**
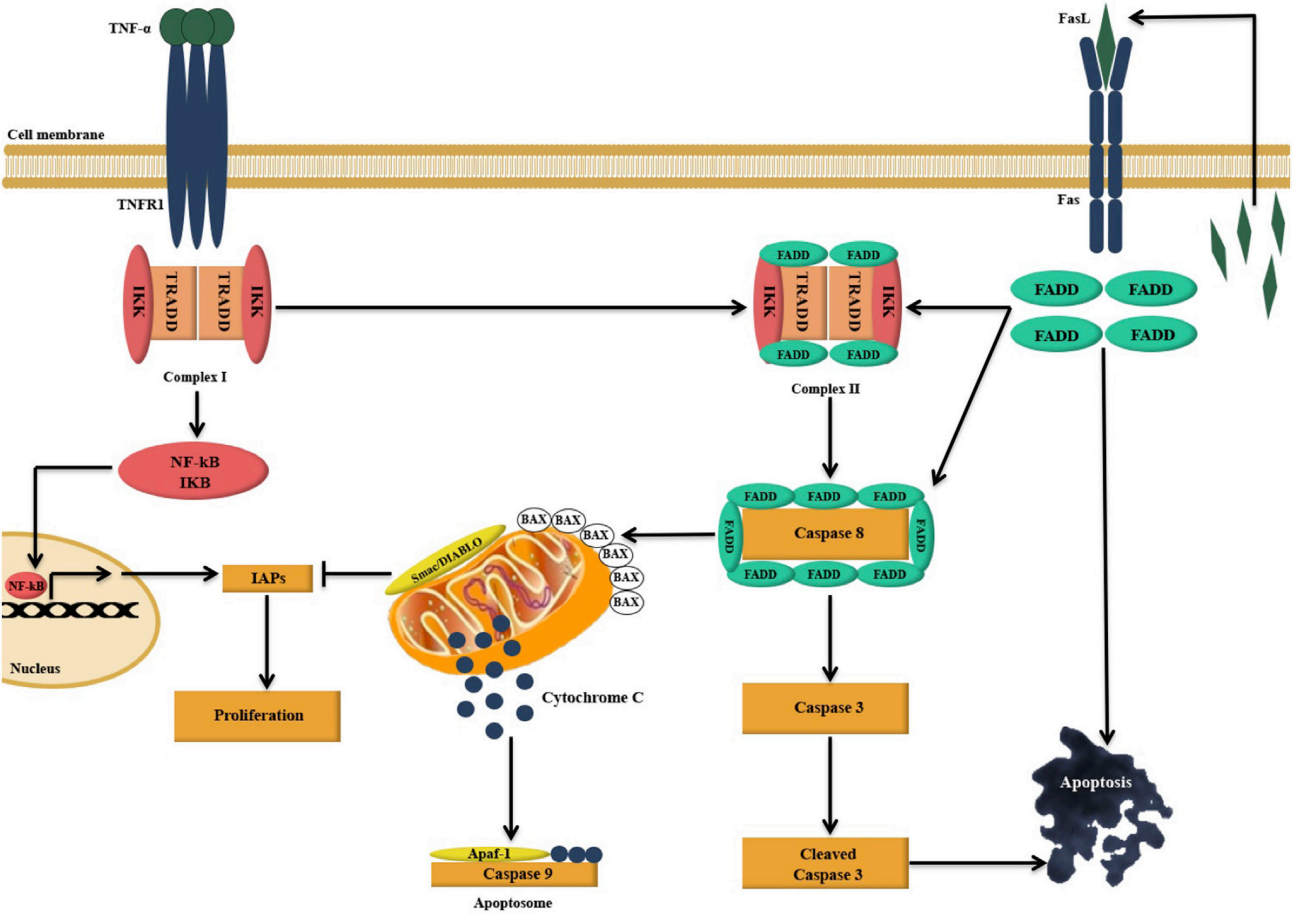
**Illustration of the likely apoptosis pathway in action following C1q binding to SKOV3 cells**. Exogenous treatment of SKOV3 cells with C1q and individual globular head modules can induce upregulation of TNF-α and Fas. TNF-α stimulation triggers two parallel, but contrasting, pathways: the pro-apoptotic caspase (15) and the anti-apoptotic NFκB-IκB inhibitor of apoptosis proteins (IAP) pathway (16). The cell survival or death, however, depends upon interactions among the components of these two pathways as well as other factors such as mitochondrial dysfunction (15, 16). TNF-α can bind to TNF type I receptor (TNFR1), which is then internalized to first form a complex with TNFR1-associated DEATH domain, receptor-interacting protein 1 (RIP1), TNF receptor-associated factor 2 (TRAF2), and cellular inhibitor of apoptosis protein-1 (c-IAP1) (complex I), triggering the upregulation of nuclear factor-κB (NF-κB). A second complex (complex II) is later formed when complex I bind to Fas-associated protein with death domain (FADD), which is recruited upon activation of Fas (15, 17). Complex II subsequently causes downstream activation of apoptosis signal by activating caspase cascade, resulting in the cleavage of initiator caspase 8 and effector caspase 3 leading to apoptosis (15, 17–19). However, NF-κB upregulation may intersect apoptotic pathway as it can promote the induction of various cellular apoptosis inhibitors such as TRAF1, TRAF2, cIAP-1, c-IAP-2, and X-linked inhibitor of apoptosis protein (XIAP) (15, 20).

We report here, for the first time, that exogenous treatment with human C1q and recombinant globular head modules ghA, ghB, and ghC caused apoptosis in an ovarian cancer cell line, SKOV3. This was evident from a significant increase in the number of Annexin V-positive cells that were examined *via* immunofluorescence microscopy and FACS, presence of cleaved caspase 9 (data not shown) and 3 *via* western blot, and transcriptional upregulation of pro-apoptotic markers, such as TNF-α, Bax, and Fas as illustrated in Figure 9. Based on these observations, it appears likely that C1q and individual globular head modules can induce apoptosis in SKOV3 cells *via* TNF-α-induced apoptosis pathway. However, other pathways could potentially be involved.

During early apoptosis, the integrity of the cell membrane is lost and phosphatidylserine (PS) is exposed for Annexin V binding (21). A significant number of SKOV3 cells were stained positive for Annexin V, when examined under immunofluorescence microscope, suggesting that the cell membrane integrity was no longer intact as Annexin V labeled with FITC was able to bind to PS, which is usually present on the intracellular leaflet of the healthy cells. However, it is translocated to the outer leaflet during apoptosis. Hence, it provided the first evidence that SKOV3 cells were undergoing apoptosis following the treatment with C1q or globular heads for 24 h (Figure 3). Trypan blue exclusion (Figure 2A) and MTT assays (Figure 2B), as well as FACS analysis (Figure 4) of treated SKOV3 cells, validated C1q-induced apoptosis of SKOV3 cells. Thus, we investigated if the levels of pro-apoptosis factors such as Fas and Bax were altered following C1q treatment of SKOV3 cells. This subsequently led us to examine the TNF-induced apoptosis pathway.

Tumor necrosis factor-α is a prototypical member of pro-inflammatory cytokine family that performs various roles in inflammation and cell death (22). Members of the TNF family are associated with a wide range of immune processes and play an important role in cancer immune surveillance For example, TNF-α can mediate apoptosis selectively in tumor cells *via* death receptors involving TRAIL/FasL ligands (23). Following treatment of SKOV3 cells with C1q or globular head modules, the gene expression of TNF-α was upregulated at 6 h (C1q) and 12 h (C1q, ghA, ghB, and ghC; Figure 5A), which underwent apoptosis by 24 h. Interaction of TNF-α with its receptor TNFR1 can cause upregulation of NF-κB, as was the case in our study (Figure 5B). Fas mRNA upregulation is observed, as expected (Figure 6A), suggesting that Fas, a receptor for FasL and a member of TNF receptor family, mediated apoptosis crosstalk. Intriguingly, Bax, a marker of dysfunctional mitochondria, was also seen to be transcriptionally upregulated (Figure 6B). Bax acts as a signal for the release of apoptogenic factors such as cytochrome *c*, Smac (second mitochondria-derived activator of caspase)/DIABLO (direct inhibitor of apoptosis protein-binding protein), apoptosis-inducing factor, Omi/HtrA2, or endonuclease G from the mitochondria. Release of Smac/DIABLO and Omi/HtrA2 into cytosol has been shown to promote caspase activation by antagonizing the IAPs, which are formed following the NF-κB upregulation (16). This suggests that Bax upregulation in our study may have negated the effects of NF-κB signaling pathway to cause inhibition of apoptosis. In addition, Bax can also trigger caspase activation by forming a cytochrome *c*/apoptotic protease activating factor 1/caspase 9-complex, which can further cause cleavage of caspase 3, or by proceeding via caspase-independent death effectors (16, 24–26).

SKOV3 cell line has previously been reported to have a dysfunctional apoptosome, which causes the activation of caspase 9, to some extent, to further cleave caspase 3 (27). This is supported by our results since activation of caspase 9 was observed (data not shown), which was unable to activate caspase 3, suggesting that cleaved caspase 3 (Figure 7B) could not have arisen exclusively due to mitochondrial pathway and validated a crosstalk between intrinsic and extrinsic pathways. This observation appears to suggest that C1q-induced apoptosis in SKOV3 cells had taken place, primarily *via* the extrinsic pathway.

Ovarian cancer cells, including SKOV3 cancer cell line, exhibit constitutive activation of Akt/mTOR survival pathway, thus protecting the cancer cells from apoptosis (28). mTOR, a serine–threonine kinase, upon activation by Akt, forms two distinct multi-protein complexes, mTOR complex 1 (mTORC1) and mTOR complex 2 (mTORC2). mTORC1 then phosphorylates p70S6K, an essential protein required for cell proliferation and G1 cell cycle progression (14, 29). Hence, we investigated if treatment of SKOV3 cells with C1q and globular heads modulated the gene expression of mTOR as well as components of mTORC1 [regulatory-associated protein of mTOR (RAPTOR)] (30) and mTORC2 [rapamycin-insensitive companion of mTOR (RICTOR)] (31). Following the treatment with C1q and globular head modules, the mRNA levels of mTOR, RICTOR, and RAPTOR were significantly downregulated at 6 h (Figure 8A). We also used immunofluorescence microscopy to show that there was a dramatic downregulation of mTOR at the protein level following treatment with C1q, ghA, ghB, or ghC (Figure 8B). These observations further supported that C1q and globular head modules not only induced apoptosis signaling pathway but also downregulated the cell survival regulators.

In conclusion, exogenous treatment of SKOV3 cells with C1q and recombinant globular head modules (ghA, ghB, and ghC) induce upregulation of TNF-α and Fas and induce apoptosis either independently, or *via* caspase cascade, in addition to downregulating the mTOR cell survival pathways. C1q has been shown to promote proliferation in the tumor microenvironment without involvement of complement activation C1q can act as an extracellular matrix protein, thus facilitating tumor growth and invasion. Experiments using C1q gene-deficient mice have also suggested that the frequency of proliferating tumor cells is considerably reduced *in vivo* (6). In another study, an opposite complement-independent function of C1q has been reported. C1q can induce apoptosis in DU145 prostate cancer cells *via* activation of WOX1, a tumor suppressor and a pro-apoptotic protein, and destabilize the cell adhesion (32). We report here that SKOV3 ovarian cancer cells are susceptible to apoptosis induction by C1q *via* TNF pathway.

C1q has a number of diverse functions including clearance of immune complexes, pathogens, and apoptotic and necrotic cells. Being the key molecule of the classical pathway of the complement system, it is a potent link between innate and adaptive immunity. However, C1q is a primordial innate immune molecule. The identification of C1q family (33, 34), based on the presence of gC1q domain, has given rise to the notion that C1q has several complement-independent functions (3). Indeed, C1q is involved in cellular differentiation, immunologic tolerance, pathology of pregnancy, developmental synaptic pruning, and extensive dialog with extracellular matrix proteins (4). Based on the jellyroll topology of the gC1q domain (33), it has also become evident that C1q is potentially a prototypical (ancestral) molecule of TNF family members, hence, existence of a C1q–TNF superfamily (1). Thus, of several overlaps of functions between C1q and TNF family members, the current study adds an additional layer of criss-cross mechanism, establishing the ability of C1q to regulate TNF-α in cancer, leading to apoptosis of the target cells. In this context, a cytokine-like property of C1q has been proposed recently (35). It is perhaps no co-incidence that the recombinant fragments of the gC1q domain of human C1q (ghA, ghB, and ghC) are able to induce apoptosis in SKOV3 cells.

It will be interesting to correlate the levels of C1q in serum and AF with the diagnosis and classification of the severity of the ovarian cancer. Due to the lack of a genuine mouse model of ovarian cancer, it will be pertinent to further establish the susceptibility and/or resistance of primary ovarian cancer cells to C1q-mediated induction of apoptosis.

## Author Contributions

AK carried out key experiments with technical help from SS and VM; FA and EK provided important reagents; AP provided technical and scientific supervision to the project; and UK led the work and, together with AK, wrote the manuscript.

## Conflict of Interest Statement

The authors declare that the research was conducted in the absence of any commercial or financial relationships that could be construed as a potential conflict of interest.
